# Rheological Behavior of *Glycyrrhiza glabra* (Licorice) Extract as a Function of Concentration and Temperature: A Critical Reappraisal

**DOI:** 10.3390/foods9121872

**Published:** 2020-12-15

**Authors:** Laleh Nasiri, Mohsen Gavahian, Mahsa Majzoobi, Asgar Farahnaky

**Affiliations:** 1 Department of Food Science and Technology, College of Agriculture, Shiraz University, Shiraz 71444-65186, Iran; laleh_nasiri@yahoo.com; 2 Department of Food Science, National Pingtung University of Science and Technology, Pingtung 91201, Taiwan; 3 Biosciences and Food Technology, School of Science, RMIT University, Bundoora West Campus, Melbourne, VIC 3083, Australia; mahsa.majzoobi@rmit.edu.au (M.M.); asgar.farahnaky@rmit.edu.au (A.F.)

**Keywords:** food rheology, non-Newtonian behavior, shear-thinning, plant extract, physical properties, food processing

## Abstract

In the present study, rheological properties of twelve different licorice root extracts were evaluated using a rotational viscometer as a function of soluble solids content (15–45 °Bx) and temperature (30–70 °C). Response Surface Methodology was used to understand the relationships between the parameters. The experimental data were then fit into mathematical models. The results, for the first time, revealed that the licorice solutions had non-Newtonian shear-thinning behaviors with flow behavior indexes of 0.24 to 0.91, depending on the licorice extract samples, temperature, and °Bx. These observations were different from those reported in the literature and the present study elaborated on reasons for such observations. Further, the shear-thinning behavior generally increased by increasing the °Bx and decreasing the temperature. In addition, the power-law model was found to be suitable for predicting the experimental data. The newly revealed information can be particularly important in designing the unit operations for licorice extract processing.

## 1. Introduction

Licorice (*Glycyrrhiza* spp.) is one of the economically important medicinal plants from Leguminosae family. The *Glycyrrhiza* genus consists of several species but only three of them, that is, *Glycyrrhiza glabra* L., *Glycyrrhiza inflata* Bat., and *Glycyrrhiza uralensis* Fisch., are officially certified by China Pharmacopoeia Committee as medicinal plants that can be used for the production of pharmaceuticals [1]. In addition, the extracts obtained from this natural ingredient possesses anti-tumor, anti-inflammatory, and antioxidant activities and has been used in the food industry for producing seasoning agents, sweetening agents, drinks, confectioneries, and functional foods [2,3,4,5]. It is also reported that the extract of this plant possesses interfacial and emulsifying properties [6]. Such health-promoting effects are believed to be correlated with the chemical composition (e.g., bioactive compounds) of this plant [7]. The chemical composition of licorice extracts varies depending on serval parameters including the plant species, growing condition, harvesting time, and extraction procedure [8,9]. However, relatively high concentrations of valuable bioactive components, including flavonoids (e.g., 0.2–2.5% Liquiritin), triterpenoid saponins (e.g., glycyrrhizin), coumarins, phenols, and polysaccharides (e.g., glycyrrhiza polysaccharides), are among the reasons for considering licorice extracts as “essential herbal medicine” [10]. Generally, the commercial extracts of this herb are found in liquid, concentrate, or powder forms [11]. The typical process of licorice extract production consists of four consecutive unit operations including grinding the herb roots, mixing with water (often at a ratio of one-to-five), extracting licorice solution usually in an extraction unit, such as hot stirred tank and finally concentration to a about 45 °Bx. [11]. The concentrate may be later converted to powder or be diluted with water before using as food ingredients.

Understanding the flow behavior of the concentrates and diluted extracts of the licorice can provide crucial information for quality control, sensory evaluation, transportation process (e.g., pumping), unit operations, and process design [12]. Designing a process without such important information may result in many issues in the production line such as difficulty to pump the licorice extract or miscalculation of the mixing conditions to produce licorice-based products. Hence, the present study was carried out to explore the rheological behavior of licorice extracts at various concentrations temperatures. Moreover, this study evaluated the applicability of the previously proposed mathematical models, i.e., Arrhenius and power-law models, for predicting the flow behavior of licorice extracts.

## 2. Materials and Methods

### 2.1. Sample Preparation

Twelve commercial licorice extracts with the degrees Brix of about 45° were obtained from the root of various Iranian *Glycyrrhiza glabra* samples using the commercial production line of an extraction company (Rishmak, Shiraz, Iran). The extracts were then converted to powders (sample codes 1–12). The concentrates obtained from the commercial production line were then diluted by distilled water to obtain samples with 15, 20, 30, 40, and 45 °Bx. The °Bx of samples were double-checked in triplicates using a laboratory refractometer (Abbe, Germany).

### 2.2. Chemical Analysis of Samples

The chemical compositions of the fresh extracts obtained from licorice, that is, before the drying process, were assessed. In this regard, the moisture, ash, glycyrrhizin, including glycyrrhizin insoluble in cold water and glycyrrhizin insoluble in hot water, and gums and starch contents were determined based on standard procedures described in the literature [13]. All the tests were performed in triplicates.

### 2.3. Rheological Studies

A rotational viscometer DV-II+Pro (Brookfield Engineering Inc., Stoughton, MA, USA) was employed to perform the rheological studies at various degrees Brix (15, 20, 30, 40, and 45 °Bx) and temperatures (30 to 70 °C). The viscometer used in the present study was equipped with a CP51 geometry (cone and plate). These tests were performed using a closed cone and plate geometry supplied by the manufacturer to minimize moisture loss during the experiment. In each experiment, 0.5 mL of licorice extract was placed in the sample compartment. Afterward, a programmed shear rate was applied to the sample. The program consists of a linear increase in shear rate from 0.77 to 140 s^−1^ in 180 steps after temperature equilibrium. The sample holder was cleaned each time and new sample was placed there for the next experiments. In addition, the calibration was performed before each set of tests. The graphs of shear stress versus shear rate were analyzed using V3.1 Rheocalc software to obtain the rheological indexes. This was performed by the computer software provided by the supplier. In addition, the power-law model (Equation (1)) was used to compute the flow behavior index and consistency coefficient values of the extracts [14].

(1) $$\tau =k{\gamma {}^{\circ}}^{n}$$

where *γ*° is the shear rate (s^−1^), *k* is the consistency coefficient (mPa·s), *n* is the dimensionless flow behavior index, and *τ* is the shear stress (mPa).

### 2.4. The Temperature Dependency of Extracts Rheological Behavior

The rheological data obtained at 30, 40, 50, 60, and 70 °C were utilized to evaluate the temperature dependency of the consistency coefficient. In this regard, the experimental data were fit into the Arrhenius model according to Equation (2) [15].

(2) $$k=k_{t}\times e^{\left(\frac{E_{a}}{R\times T}\right)}$$

where *E_a_* the activation energy (J·mol), *k_t_* is the consistency coefficient at a reference temperature; that is, proportionality constant (Pa·s), *T* is the absolute temperature (*K*), and *R* the universal gas law constant (J·mol·K).

### 2.5. Statistical Analysis

The theological characteristics of licorice extracts were assessed through a linear regression method to achieve the minimum possible values of the sum of squares as performed by the software provided by Brookfield company (Rheocal, Version 3.1, Brookfield Engineering, Middleborough, MA, USA). In addition, the regression coefficients (R^2^) and model equations were obtained. Further, the analysis of variance (ANOVA) was used to detect significant differences between the data at the significance level of 0.01. To achieve this, the SPSS 13.0 software was utilized. Furthermore, the D-optimal Response Surface mode of version 6.0.2 of Design-Expert software (Stat-Ease Inc., Minneapolis, MN, USA) was utilized to model the experimental data and to estimate nonlinearity relationships between the parameters according to Response Surface Methodology (RSM) [16].

## 3. Results and Discussion

### 3.1. Chemical Composition of Licorice Extracts

Chemical compositions of twelve fresh licorice extracts (sample codes 1 to 12), that is fresh extracts after the extraction process and before the drying process, are presented in Table 1. It was previously explained that the extraction methods, plant origin, growth, and harvesting conditions can affect the composition of the plant extracts [8,9,17]. A previous report mentioned that a licorice extract sample collected in Italy contains 42.0% gums and starch, 2.3%, 2.1% sucrose, 5.4% glycyrrhizin, 13.4% moisture content, and about 29.1% insoluble and other compounds [12]. The moisture and ash contents of the samples in the present study ranged between 53.28–62.95% and 7.44–9.63%, respectively. The differences in ash contents of the samples are mainly attributed to plant species, growth conditions, and processing parameters such as the extent of washing of the starting raw materials as well as the design of decantation and filtration stages. Gums and starch are mainly polysaccharides that precipitate in the presence of ethanol. Gums and starch content of samples were in the range of 21.93–32.45%.

### 3.2. Rheological Properties of Extracts

#### 3.2.1. Flow Behaviors

**Figure 1:**
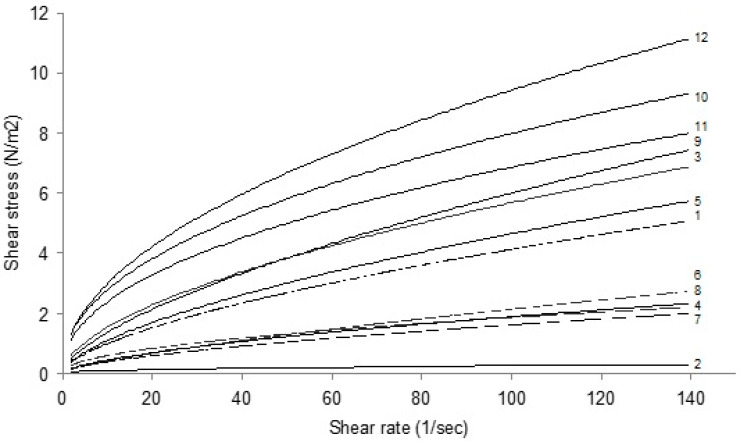
Shear stress versus shear rate of twelve licorice extract solutions with the degrees Brix of 30 (sample numbers 1 to 12) at 60 °C.

The slope of shear stress–shear rate curves of all twelve samples tested with degrees Brix decreased as the shear rate increased at all the temperatures (Figure 1). It reveals that the viscosity of licorice extract samples declines with shear rate, that is, shear-thinning behavior. This trend was observed for all samples regardless of the degrees Brix and temperature of the licorice extracts (graphs not shown). Large differences were observed between the samples at the same concentration and temperature (e.g., between sample codes 2 and 12 at 30 °Bx at 60 °C).

#### 3.2.2. Effects of Temperature and Degrees Brix on Licorice Viscosity

The results of the power-law model for analyzing the shear stress–shear rate data revealed the rheological indexes of various samples at various degrees Brix and temperatures. The consistency coefficients of twelve licorice extract samples at 30 and 60 °C are presented in Table 2.

**Figure 2:**
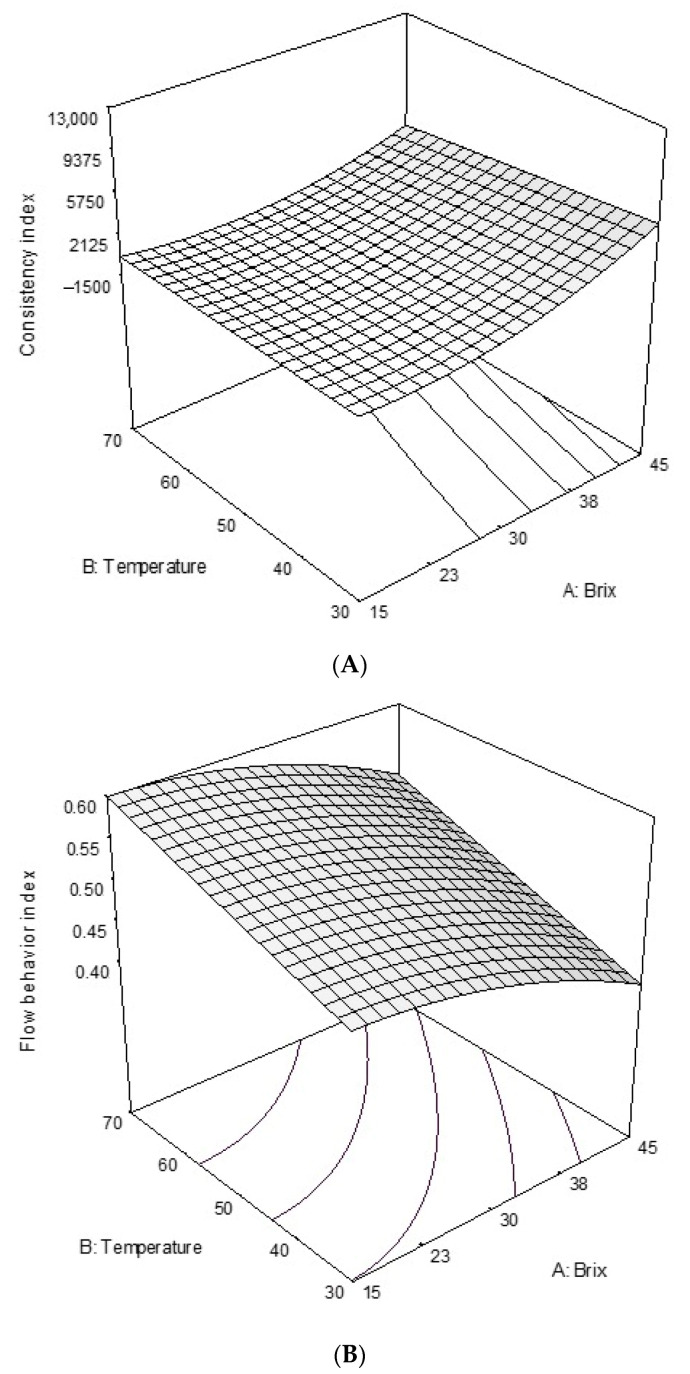
(**A**) The effects of temperature and degrees Brix on the consistency index (cP) of licorice solutions at shear rate of 50 sec^−1^ and (**B**) The effects of temperature and degrees Brix on the flow behavior index of licorice extract solutions.

According to the results, the consistency coefficient increased with increasing the degrees Brix for all samples. Therefore, degrees Brix had a significant effect on this parameter and there was a direct relationship between the soluble solid contents and the viscosity at a constant temperature. For example, the consistency coefficient of sample #4 at 30 °C was 31.10, 289.65, 499.35, 2947.50, and 7781 mPa·s when the degrees Brix was 15, 20, 30, 40, and 45, respectively (Figure 2). This is due to the dilution of solid materials resulting in a decrease of solid–solid and water–solid interactions which is in agreement with a previous report [18]. The same trend was observed for all samples under all test conditions in terms of degrees Brix and temperature. Moreover, an inverse relationship between viscosity and temperature was observed, that is, the viscosity decreased with increasing temperature. This observation is because of the higher molecular movement at elevated temperatures as explained in the literature [14].

#### 3.2.3. Flow Behavior Index of Extracts

Flow behavior index (*n*) of the samples that were obtained from the power-law equation for all Brix and temperatures (30 and 60 °C) are presented in Table 3. With no exception, the flow behavior indexes of all tested samples were less than one, confirming the shear-thinning behavior of the licorice extracts. For example, this value for the twelve samples ranged from 0.26 to 0.64 for Brix 30 at 30 °C. According to the data reported in the present study, non-Newtonian flow behaviors were observed for all samples at different concentrations and temperatures.

In an earlier study, the rheological properties of water extracts of licorice roots were assessed by a Brookfield viscometer at a temperature range of 10 to 60 °C and soluble solids 3 to 50 degrees Brix [19]. This author reported a Newtonian flow behavior for licorice extract for all concentrations which is different from those observed in the present study. In the present study, the experiments were performed using a cone and plate geometry at a wide range of shear rates, i.e., up to 140 sec^−1^ (Figure 1). However, in the research carried out by Maskan [20] a Brookfield viscometer Model RVT (Brookfield Engineering Laboratories Inc., Stoughton, MA, USA) with the spindle No. 2 emerged in a 600 mL beaker containing the licorice sample had been used. In principle, well-defined geometries such as cone and plate or parallel plates are the geometries of choice for the study of shear rate dependency of solutions and Brookfield viscometer Model RVT with its limitations in terms of using a spindle submerged in a beaker with a large shear rate distribution across the sample does not seem to be suitable. On the other hand, due to the equipment limitations only speeds of 10, 20, 50, and 100 rpm, i.e., shear rates of up to 27 sec^−1^ was tested by Maskan [20]. Such an approach may undermine the validity of the results extracted from the experimental data in that old work. Gabriele et al. (2001) used a rheological-based approach to study the flow behavior of licorice samples with a moisture content of 13% *w*/*w* during the extrusion process. These authors employed an oscillatory rheometer equipped with a temperature control unit and obtained the flow curves of the licorice extract from the elaboration of the creep data. According to the authors, a linear relationship between shear rate and viscosity was reported for licorice powder. These different observations, compared to the new information released in the present study, are mainly related to sample specification. Moreover, it should be noted that selecting appropriate systems for studying the physical properties of materials, such as rheological behavior, is a key to collect valid data [20]. Hence, advances in technology may result in more precise data and improve our understanding of science. This could be the case for differences between the observation in the present study with those reported in the past century by Maskan [20]. In addition, as explained in previous sections of the manuscript, it is known that the extraction method and other parameters (e.g., plant growth conditions) may affect the chemical composition of the extract, resulting in a different rheological property [21,22,23].

### 3.3. Response Surface Methodology Results

RSM results explain the changes in rheological indexes and viscosity as affected by temperature and degrees Brix for various samples (Figure 2). According to the data obtained in the present study, all extracts at all the studied conditions had non-Newtonian shear-thinning behaviors; that is, the flow behavior indexes were less than 1 for 15–45 °Bx. In addition, the consistency index increased with degrees Brix of sample and decreased with an increase in temperature. Furthermore, non-linear relationships between the dependent and independent parameters for both consistency and flow behavior indexes were observed. These observations were in line with those documented in the literature [24]. From the 3-D graphs obtained from surface response methodology (Figure 2), it can be concluded that the effect of Brix on consistency or flow behavior indexes was greater than that of temperature within the concentration and temperature ranges examined in the present study.

### 3.4. Activation Energy

**Figure 3:**
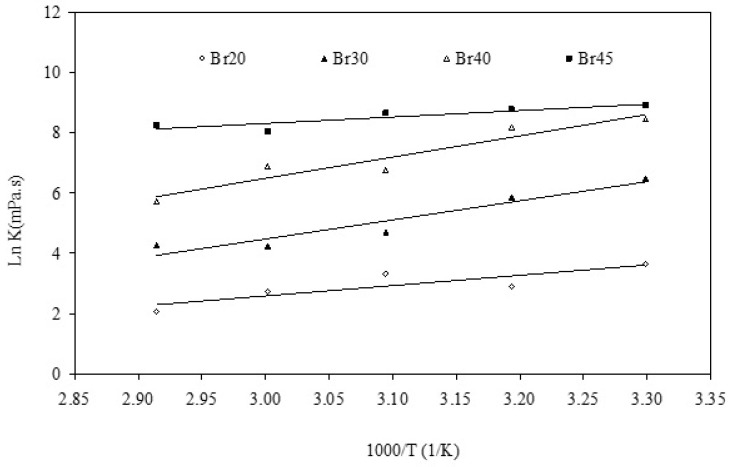
Ln K versus 1/temperature for licorice extract samples with various °Brix (Br).

The Arrhenius type equation was used to study the temperature-viscosity data (Figure 3) as explained in Equation (2). As a result of this investigation, the activation energy values were calculated. According to the results, for °Brix of 20, Activation energy (*E_a_*), the proportionality constant (*K_t_*), and R-square were found to be 29,053 (KJ/mol), 4.67 × 10^−3^ (mPa·s), and 0.71, respectively. Further, when the °Brix was 30, *E_a_*, *K_t_*, and R-square were 52,713 (KJ/mol), 4.84 × 10^−7^ (mPa·s), and 0.91, respectively. However, when the °Brix was 40, *E_a_*, *K_t_*, and R-square were 58,788 (KJ/mol), 4.06 × 10^−7^ (mPa·s), and 0.91, respectively. At the highest degrees of the Brix, it was observed that *E_a_*, *K_t_*, and R-square were 17,798 (KJ/mol), 7.70 (mPa·s), and 0.79, respectively.

The results showed that the Arrhenius equation is an appropriate model to describe temperature dependency of licorice extract viscosity and the correlation coefficients were found to be in a range of 0.71 to 0.91. Moreover, it was observed that with increasing the degrees Brix (up to 40) the calculated activation energy increased. Therefore, the temperature had a more profound effect on licorice root extract viscosity when the sample had a greater value of soluble solids content. This finding can be particularly useful in the industry where the viscosity of the in-process sample can be adjusted (e.g., by a small change in the temperature) to meet the equipment, such as pump, requirements.

## 4. Conclusions

For the first time, this study revealed that all licorice root extract solutions exhibited shear-thinning non-Newtonian behavior at all temperatures and degrees Brix. The results obtained in this research disagreed with the previously reported Newtonian behavior for licorice extracts which could be due to the variations in the chemical composition of the root extracts and rheological study methodology. In addition, it was revealed that the viscosity of the extracts originated from different regions varies significantly. The information provided in the present study can be used for amending the process design and unit operation conditions of the related processes in the food and pharmaceutical industries.

## Figures and Tables

**Table 1:** The chemical composition (dry weight basis) of twelve licorice extract samples (sample codes of 1–12) that were harvested at various seasons and produced at commercial licorice processing line.

Licorice Sample Code	Moisture (%)	Ash (%)	Gums and Starch (%)	Glycyrrhizin (%)	Insoluble in Cold Water (%)	Insoluble in Hot Water (%)
1	53.28 ± 0.35 ^a^	8.38 ± 0.27 ^cd^	23.79 ± 0.73 ^c^	16.39 ± 0.23 ^bc^	20.82 ± 0.84 ^g^	0.87 ± 0.22 ^ab^
2	62.74 ± 0.13 ^gh^	9.63 ± 0.06 ^e^	21.93 ± 0.73 ^a^	22.77 ± 1.01 ^d^	3.34 ± 0.52 ^a^	1.38 ± 0.59 ^abc^
3	61.77 ± 0.08 ^de^	8.15 ± 0.36 ^abcd^	31.37 ± 0.13 ^fg^	16.35 ± 0.32 ^bc^	13.52 ± 0.75 ^de^	1.54 ± 0.84 ^bcd^
4	61.83 ± 0.13 ^def^	8.55 ± 0.09 ^d^	25.46 ± 0.65 ^d^	17.31 ± 0.30 ^c^	50.11 ± 0.49 ^c^	2.69 ± 0.52 ^d^
5	62.40 ± 0.54 ^fgh^	7.47 ± 0.47 ^a^	29.15 ± 0.71 ^e^	17.77 ± 0.35 ^c^	12.38 ± 0.17 ^cd^	2.21 ± 0.38 ^bcd^
6	62.95 ± 10.0 ^h^	8.56 ± 0.29 ^d^	23.43 ± 0.83 ^bc^	22.63 ± 1.69 ^d^	8.38 ± 0.50 ^b^	0.48 ± 0.16 ^ab^
7	61.51 ± 0.33 ^d^	8.30 ± 0.21 ^bcd^	23.66 ± 0.37 ^c^	15.91 ± 1.48 ^abc^	10.61 ± 0.39 ^c^	1.45 ± 0.73 ^abc^
8	62.17 ± 0.03 ^efg^	7.96 ± 0.35 ^abcd^	22.05 ± 0.72 ^ab^	14.99 ± 0.18 ^ab^	18.56 ± 0.24 ^f^	0.66 ± 0.25 ^ab^
9	60.34 ± 0.36 ^c^	7.64 ± 0.37 ^ab^	25.65 ± 0.04 ^d^	14.06 ± 1.15 ^a^	23.74 ± 1.09 ^h^	0.82 ± 0.16 ^ab^
10	60.63 ± 0.16 ^c^	7.74 ± 0.13 ^abc^	32.45 ± 0.23 ^g^	16.23 ± 0.27 ^bc^	18.28 ± 1.52 ^f^	0.30 ± 0.13 ^ab^
11	61.52 ± 0.12 ^d^	7.44 ± 0.34 ^a^	30.54 ± 0.23 ^ef^	16.63 ± 0.47 ^bc^	50.18 ± 0.41 ^f^	0.30 ± 0.13 ^ab^
12	58.56 ± 0.14 ^b^	8.09 ± 0.12 ^abcd^	29.62 ± 0.14 ^e^	17.51 ± 0.26 ^c^	14.98 ± 0.34 ^e^	0.17 ± 0.05 ^a^

The similar letters refer to no significant differences at 0.05 level.

**Table 2:** The consistency coefficient, k, (mPa·s) of twelve licorice extract samples harvested at various seasons and produced using the same commercial licorice processing line as a function of degrees Brix at 30 and 60 °C.

Licorice Sample Code	°Brix				
	15	20	30	40	45
	30 °C				
1	11.39 ± 1.50 ^a^	35.75 ± 3.35 ^a^	670.45 ± 121.05 ^b^	1010.80 ± 97.25 ^b^	3985.00 ± 36.00 ^d^
2	27.62 ± 4.58 ^bc^	29.52 ± 7.11 ^a^	46.95 ± 2.75 ^a^	85.25 ± 0.25 ^a^	139.85 ± 0.85 ^a^
3	42.90 ± 2.30 ^d^	206.05 ± 2.95 ^e^	2887.50 ± 173.50 ^d^	5411.50 ± 404.50 ^i^	ND
4	31.10 ± 0.10 ^cd^	280.65 ± 11.15 ^f^	499.35 ± 33.05 ^b^	2947.50 ± 101.50 ^f^	7781.00 ± 21.00 ^h^
5	83.80 ± 16.80 ^e^	160.95 ± 4.05 ^c^	610.95 ± 34.95 ^b^	4751.50 ± 63.50 ^h^	7504.50 ± 47.50 ^g^
6	15.00 ± 3.30 ^ab^	37.85 ± 2.15 ^a^	652.90 ± 19.40 ^b^	4740.00 ± 63.50 ^h^	7440.50 ± 105.50 ^g^
7	84.90 ± 0.60 ^e^	101.25 ± 0.45 ^b^	333.50 ± 31.10 ^ab^	1866.50 ± 9.50 ^d^	3086.80 ± 61.77 ^c^
8	18.40 ± 0.70 ^abc^	40.15 ± 0.45 ^a^	264.90 ± 27.70 ^ab^	3728.50 ± 29.50 ^g^	5220 ± 46.35 ^e^
9	19.55 ± 0.45 ^abc^	208.00 ± 6.85 ^e^	441.00 ± 31.40 ^ab^	1522.50 ± 90.50 ^c^	2847.50 ± 125.50 ^b^
10	143.70 ± 1.30 ^g^	421.65 ± 6.85 ^g^	1454.50 ± 105.50 ^c^	8019.00 ± 40.00 ^j^	ND
11	87.10 ± 1.80 ^e^	188.85 ± 0.75 ^d^			
12	115.70 ± 0.60 ^f^	197.50 ± 13.50 ^de^			
	60 °C				
1	5.80 ± 0.840 ^a^	15.06 ± 2.56 ^a^	230.90 ± 3.70 ^e^	491.55 ± 11.15 ^c^	4637.50 ± 159.50 ^f^
2	4.82 ± 4.20 ^a^	10.17 ± 1.73 ^a^	29.30 ± 3.60 ^a^	36.30 ± 4.80 ^a^	50.30 ± 6.00 ^ab^
3	10.86 ± 4.94 ^a^	59.90 ± 6.70 ^c^	315.95 ± 3.15 ^f^	5863.50 ± 8.50 ^h^	ND
4	22.95 ± 1.45 ^a^	75.55 ± 2.95 ^d^	175.20 ± 14.30 ^d^	206.96 ± 0.64 ^b^	1847.50 ± 122.50 ^d^
5	54.70 ± 7.20 ^b^	124.60 ± 12.80 ^e^	212.00 ± 21.50 ^e^	3779.50 ± 120.50 ^f^	7343.00 ± 689.00 ^g^
6	4.67 ± 0.47 ^a^	15.10 ± 2.10 ^a^	69.95 ± 1.15 ^b^	969.85 ± 4.25 ^d^	3124.00 ± 223.00 ^e^
7	13.70 ± 1.50 ^a^	60.80 ± 5.20 ^c^	70.80 ± 0.20 ^b^	408.15 ± 7.05 ^c^	544.70 ± 8.40 ^b^
8	25.25 ± 1.35 ^a^	57.50 ± 0.70 ^a^	103.45 ± 0.85 ^c^	476.20 ± 25.10 ^c^	4738.00 ± 12.20 ^f^
9	14.96 ± 8.54 ^a^	39.35 ± 0.15 ^b^	408.50 ± 20.00 ^g^	1005.50 ± 17.50 ^d^	1132.00 ± 77.14 ^c^
10	49.00 ± 25.30 ^b^	78.10 ± 0.60 ^d^	959.30 ± 0.50 ^i^	4381.00 ± 4.00 ^g^	ND
11	20.45 ± 3.04 ^a^	53.05 ± 2.65 ^c^	824.05 ± 22.55 ^h^	5839.00 ± 138.00 ^h^	ND
12	18.20 ± 1.10 ^a^	20.15 ± 2.35 ^a^	941.90 ± 7.50 ^i^	2389.00 ± 98.00 ^e^	3437.50 ± 80.00 ^e^

The similar letters refer to no significant differences at 0.05 level. ND: not detected.

**Table 3:** Flow behavior indexes, *n*, of twelve licorice extract samples harvested at various seasons and produced using the same commercial licorice processing line as a function of degrees Brix at 30 and 60 °C.

Licorice Sample Code	°Brix				
	15	20	30	40	45
	30 °C				
1	0.47 ± 0.05 ^a^	0.71 ± 0.02 ^hi^	0.49 ± 0.04 ^d^	0.63 ± 0.02 ^f^	0.51 ± 0.01 ^d^
2	0.78 ± 0.02 ^c^	0.77 ± 0.03 ^h^	0.78 ± 0.02 ^g^	0.79 ± 0.01 ^g^	0.80 ± 0.00 ^f^
3	0.56 ± 0.01 ^ab^	0.47 ± 0.00 ^b^	0.26 ± 0.01^a^	0.37 ± 0.01 ^bc^	ND
4	0.55 ± 0.01 ^ab^	0.34 ± 0.01 ^a^	0.39 ± 0.01 ^c^	0.38 ± 0.01 ^bc^	0.24 ± 0.00 ^a^
5	0.47 ± 0.01 ^a^	0.57 ± 0.01 ^f^	0.49 ± 0.01 ^d^	0.39 ± 0.02 ^c^	0.42 ± 0.02 ^c^
6	0.76 ± 0.01 ^c^	0.72 ± 0.01 ^i^	0.40 ± 0.01 ^c^	0.30 ± 0.01 ^a^	0.33 ± 0.00 ^b^
7	0.51 ± 0.01 ^a^	0.48 ± 0.01 ^bc^	0.45 ± 0.01 ^d^	0.42 ± 0.01 ^d^	0.43 ± 0.01 ^c^
8	0.72 ± 0.01 ^bc^	0.69 ± 0.01 ^g^	0.55 ± 0.01 ^e^	0.36 ± 0.01 ^b^	0.41 ± 0.02 ^c^
9	0.78 ± 0.01 ^c^	0.52 ± 0.00 ^d^	0.64 ± 0.01 ^f^	0.63 ± 0.01 ^f^	0.58 ± 0.01 ^e^
10	0.52 ± 0.00 ^a^	0.50 ± 0.01 ^cd^	0.47 ± 0.01 ^d^	0.32 ± 0.01 ^a^	ND
11	0.55 ± 0.00 ^b^	0.55 ± 0.01 ^ef^	0.32 ± 0.01 ^b^	0.38 ± 0.00 ^bc^	ND
12	0.49 ± 0.00 ^a^	0.53 ± 0.00 ^de^	0.52 ± 0.01 ^e^	0.50 ± 0.00 ^e^	0.43 ± 0.00 ^c^
	60 °C				
1	0.71 ± 0.06 ^b^	0.82 ± 0.03 ^f^	0.62 ± 0.01 ^d^	0.625 ± 0.01 ^g^	0.40 ± 0.01 ^c^
2	0.68 ± 0.05 ^b^	0.64 ± 0.05 ^cd^	0.46 ± 0.04 ^a^	0.82 ± 0.04 ^h^	0.89 ± 0.02 ^h^
3	0.67 ± 0.01 ^b^	0.63 ± 0.02 ^c^	0.63 ± 0.01 ^d^	0.28 ± 0.01 ^a^	ND
4	0.60 ± 0.01 ^b^	0.47 ± 0.02 ^b^	0.51 ± 0.03 ^b^	0.80 ± 0.01 ^h^	0.48 ± 0.01 ^f^
5	0.32 ± 0.02 ^a^	0.39 ± 0.02 ^a^	0.63 ± 0.03 ^d^	0.32 ± 0.01 ^b^	0.27 ± 0.01 ^a^
6	0.91 ± 0.01 ^c^	0.80 ± 0.01 ^ef^	0.74 ± 0.01 ^e^	0.49 ± 0.00 ^e^	0.39 ± 0.01 ^c^
7	0.65 ± 0.00 ^b^	0.42 ± 0.00 ^a^	0.67 ± 0.00 ^d^	0.58 ± 0.00 ^f^	0.62 ± 0.01 ^g^
8	0.66 ± 0.01 ^b^	0.77 ± 0.01 ^ef^	0.63 ± 0.01 ^d^	0.59 ± 0.00 ^f^	0.37 ± 0.01 ^b^
9	0.63 ± 0.14 ^b^	0.75 ± 0.00 ^e^	0.56 ± 0.00 ^bc^	0.47 ± 0.02 ^de^	0.42 ± 0.01 ^d^
10	0.60 ± 0.12 ^b^	0.65 ± 0.00 ^cd^	0.46 ± 0.00 ^a^	0.39 ± 0.00 ^c^	ND
11	0.38 ± 0.00 ^a^	0.69 ± 0.01 ^d^	0.46 ± 0.00 ^a^	0.27 ± 0.00 ^a^	ND
12	0.67 ± 0.01 ^b^	0.77 ± 0.02 ^ef^	0.50 ± 0.00 ^ab^	0.44 ± 0.01 ^d^	0.45 ± 0.00 ^e^

The similar letters refer to no significant differences at 0.05 level. ND: not detected.
